# Bidirectional Mendelian randomization study reveals causal relationships between polymyalgia rheumatica and serum metabolites

**DOI:** 10.1097/MD.0000000000044304

**Published:** 2025-09-05

**Authors:** Aifeng Song, Lin Kang, Saixia Cao, Yafang Wang, Huiyun Li

**Affiliations:** a Department of Rheumatology and Immunology, Inner Mongolia Autonomous Region People’s Hospital, Hohhot, China; b Department of Clinical Laboratory, Inner Mongolia Autonomous Region People’s Hospital, Hohhot, China.

**Keywords:** causal relationship, Mendelian randomization, metabolites, polymyalgia rheumatica

## Abstract

Polymyalgia rheumatica (PMR) is a prevalent chronic inflammatory disorder among the elderly, presenting diagnostic and therapeutic challenges. Existing treatments like glucocorticoids have significant side effects, and the molecular mechanisms of PMR remain unclear. This study employed a 2-sample bidirectional Mendelian randomization approach to explore the causal relationship between 1091 metabolites, 309 metabolite ratios, and PMR risk. genome-wide association study data from large-scale cohorts were utilized, including metabolite data from 8299 Europeans and PMR datasets from FinnGen R12. After identifying suitable genetic instrumental variables and conducting multiple statistical analyses, 13 metabolites were found to be significantly associated with PMR risk at a *P* <.05 significance level. Among them, 10 metabolites increased the risk, 3 decreased it, and 1 had bidirectional effects. Five androgen-related metabolites were identified as causal risk factors, while one reduced the risk. Four arachidonic acid-related metabolites and 1 ratio increased the risk, and 1-stearoyl – 2-linoleoyl – gpc (18:0/18:2) also promoted PMR development. Cysteinylglycine was found to reduce the risk, but PMR increased its metabolite levels in serum. However, the study has limitations. The genome-wide association study data mainly came from European-ancestry populations, limiting generalizability, and the functional roles and mechanistic links of some metabolites remain unclear. Despite these limitations, this study provides new insights into PMR pathogenesis and potential metabolite-based intervention targets, highlighting the need for further research in diverse ethnic groups to better understand the relationship between metabolites and PMR.

## 1. Introduction

Polymyalgia rheumatica (PMR) is a chronic inflammatory disorder characterized by severe pain and stiffness in the shoulder and pelvic girdles, predominantly affecting individuals over the age of 50. With an estimated incidence of 1 in 1000 adults annually, PMR poses significant diagnostic and therapeutic challenges due to its nonspecific symptomatology and overlap with other conditions, such as giant cell arteritis^[1–4]^(Crowson, 2017 #62). Current first-line treatment relies on prolonged glucocorticoid therapy, which is associated with substantial adverse effects, including osteoporosis, diabetes, and cardiovascular complications.^[5–7]^ Despite advances in understanding its clinical features, the molecular mechanisms driving PMR pathogenesis remain poorly defined, underscoring the urgent need to identify novel therapeutic targets.

Emerging evidence highlights a significant relationship between metabolites and PMR. Manning et al ound PMR patients have a distinct metabolite signature compared to non-inflammatory controls. Glucocorticoid treatment further changes this signature, but it doesn’t return to the control state. Some metabolites like 3-hydroxybutyrate are linked to inflammation-related symptoms, while low serum glutamine predicts high fatigue^[8]^ Iliou et al also showed that there are different metabolic profiles between active and inactive PMR. N-acetylglycoproteins and cholines can indicate disease activity, and levels of small molecules like phenylalanine and glutamine change in active disease. In brief, metabolites are important in understanding PMR, acting as potential biomarkers for disease activity and symptoms.^[9–11]^

Mendelian randomization (MR), a genetic instrumental variable approach, offers a robust framework to infer causal relationships between metabolites and disease by leveraging genetic variants associated with exposure factors as proxies.^[12]^ While bidirectional MR has been successfully applied to elucidate causal links between metabolites and some diseases,^[13–16]^ its application in PMR remains unexplored. Key questions persist: Do specific metabolites directly drive PMR pathogenesis? Addressing this bidirectional relationship is critical to disentangle cause from consequence and identify actionable therapeutic targets.

In this study, we perform a bidirectional, 2-sample MR analysis to investigate the causal relationship between serum metabolites and PMR. Utilizing genome-wide association study (GWAS) data from large-scale cohorts, we systematically assess 1400 serum metabolites for their potential causal effects on PMR risk and vice versa. Sensitivity analyses, including pleiotropy and heterogeneity testing, ensure the robustness of our findings. Our results reveal novel causal relationship between metabolites and PMR, while also identifying metabolites influenced by PMR-related genetic liability. These insights advance our understanding of PMR pathogenesis and highlight opportunities for metabolite-based interventions to improve clinical outcomes.

## 2. Methods

### 2.1. Study design

This study adopted a 2-sample bidirectional MR approach to explore the causal relationships between the 1091 metabolites and 309 metabolite ratios and the risk of polymyalgia rheumatica. First, we obtained the association data of human serum metabolites (exposure factors) from GWAS to clarify the single nucleotide polymorphism (SNP) loci associated with human metabolites and metabolite ratios and regarded them as estimates of genetic instrumental variables (IVs). Subsequently, another GWAS was conducted to obtain the association data related to polymyalgia rheumatica (outcomes) and clarify the existence of relevant SNPs. Finally, through the screened SNPs and various statistical methods, we could comprehensively judge the causal relationships between the 1091 metabolites and 309 metabolite ratios and the risk of polymyalgia rheumatica.

### 2.2. Data sources

The metabolite database was derived from one of the most extensive metabolite studies carried out by Chen et al.^[16]^ In this study, 1091 metabolites and 309 metabolite ratios were investigated in 8299 individuals of Europeans. This report ultimately pinpointed nearly 15.4 million SNPs for GWAS testing. The polymyalgia rheumatica datasets were retrieved from FinnGen R12 (https://www.finngen.fi/en), consisting of 4992 cases and 484,260 controls. The data for this study comes from public databases and does not require ethical review.

### 2.3. Genetic variation

According to the requirements of MR analysis, genetic variations serving as instrumental variables (IVs) need to meet the following 3 basic assumptions: there is a robust correlation between the instrumental variable and the exposure factor X; the instrumental variable is not related to any confounding factors that affect the association between the exposure and the outcome; and the instrumental variable does not affect the outcome Y, unless their association with the exposure X may have an indirect impact. Figure S1, Supplemental Digital Content, https://links.lww.com/MD/P870 illustrates the core assumptions and workflow of MR. To determine the IVs for the 1091 metabolites and 309 metabolite ratios, certain procedures were taken to ensure the validity of the first assumption. Initially, genetic variations were extracted using an association threshold of *P* <1 × 10^−5^, which is commonly used in MR analysis to capture greater variability when there are limited SNPs available for exposure. Subsequently, independent variants were identified through a clustering procedure implemented in R software, with a linkage disequilibrium threshold of *r*² <0.001 within a 10,000 kb. Finally, to quantitatively evaluate the strength of the selected instruments, we calculated the explained variance (*R*²) and *F*-statistic for each metabolite. Generally, a threshold of *F* >10 is recommended for further MR analysis.

### 2.4. MR statistical analysis

In this study, 5 statistical methods, namely the simple mode, weighted mode, inverse-variance weighted analysis (IVW), weighted median method, and MR-Egger regression, were used to analyze the causal relationships between 1091 metabolites and 309 metabolite ratios and the risk of polymyalgia rheumatica. All results were presented as odds ratios and their corresponding 95% confidence intervals, and a *P*-value <.05 was considered statistically significant. Statistical analysis was performed using the TwoSampleMR 0.6.12 packages in R software version 4.4.3. The leave-one-out method was used to evaluate the sensitivity of the MR results.

We conducted IVW and MR Egger tests on the data to identify evidence of heterogeneity (*P* <.05). If an instrumental variable affects the outcome through factors other than the exposure, it is considered to have pleiotropy. Pleiotropy may lead to the failure of the independence and exclusion assumptions. In our study, we used the MR-Egger intercept test and MR-PRESSO to verify variable pleiotropy and assess the robustness of our results (*P* <.05).

### 2.5. Reverse MR analysis

A reverse MR analysis was conducted to evaluate whether polymyalgia rheumatica affect the levels of candidate serum metabolites. The MR analysis was carried out according to the methods described previously.

## 3. Results

### 3.1. Determination of instrumental variables

The IVs for the 1091 metabolites and 309 metabolite ratios were generated, with the number of SNPs per variable ranging from 4 to 56. All of these IVs were effectively suitable for conducting MR analysis on the 1091 metabolites and 309 metabolite ratios, as evidenced by an *F*-statistic >10.

### 3.2. MR analyses

#### 3.2.1. Forward MR analysis

**Figure 1.**
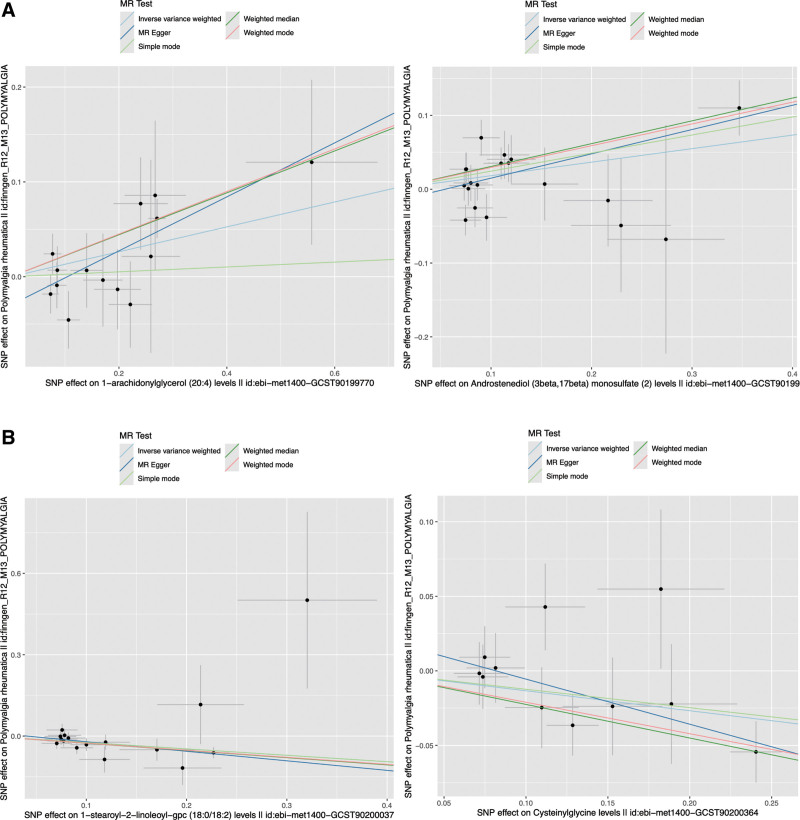
Representative scatter plots of the 5MR models for 13 screened metabolites with potential causal relationship with PMR. (A) 2 metabolites (1-arachidonylglycerol [20:4] and Androstenediol [3beta, 17beta] monosulfate [2]) increased risk of PMR signicantly; (B) 2 metabolites (1-stearoyl-2-linoleoyl-gpc [18:0/18:2] and Cysteinylglycine) decreased risk of PMR signicantly. MR = Mendelian randomization, PMR = polymyalgia rheumatic.

Five methods were utilized in the forward MR analysis, and scatter plots were generated (Fig. 1; Fig. S2, Supplemental Digital Content, https://links.lww.com/MD/P870). Employing 3 methods (IVW, weighted median, and weighted mode), causal relationships were detected between 12 metabolites and 1 metabolite ratio and polymyalgia rheumatica. Specifically, 9 metabolites and 1 metabolite ratio were found to increase the risk of polymyalgia rheumatica, while 3 metabolites could reduce this risk, as presented in Table 1.

**Table 1:** Five MR models estimate the causal relationships between 13 known metabolites and the risk of polymyalgia rheumatica and tests for heterogeneity and horizontal pleiotropy.

Metabolites	MR analysis				Heterogeneity		Pleiotropy		MR-PRESSO
	Methods	SNP (N)	OR (95% CI)	*P*	*Q* value	*P*	Intercept	*P*_MR Egger_	*P*
Epiandrosterone sulfate	IVW	15	1.09 (1.04–1.15)	.0009	12.9062	.5339	0.0075	0.4353	.7330
	MR Egger	15	1.08 (1.01–1.14)	.0315	12.2582	.5066	–	–	
	Simple mode	15	1.11 (0.92–1.35)	.290	–	–	–	–	
	Weighted median	15	1.09 (1.03–1.15)	.0029	–	–	–	–	
	Weighted mode	15	1.09 (1.03–1.15)	.0114	–	–	–	–	
1-arachidonylglycerol (20:4)	IVW	14	1.14 (1.03–1.26)	.0097	12.9747	.4498	−0.0298	0.0864	.3877
	MR Egger	14	1.33 (1.10–1.61)	.0122	9.4851	.6610	–	–	
	Simple mode	14	1.03 (0.77–1.37)	.8662	–	–	–	–	
	Weighted median	14	1.25 (1.09–1.43)	.0015	–	–	–	–	
	Weighted mode	14	1.25 (1.09–1.44)	.0080	–	–	–	–	
1-arachidonoyl-gpc (20:4n6)	IVW	16	1.07 (1.00–1.14)	.0352	12.7240	.6236	−0.0213	0.0508	.4023
	MR Egger	16	1.15 (1.05–1.26)	.0096	8.1624	.8807	–	–	
	Simple mode	16	1.11 (0.87–1.42)	.4257	–	–	–	–	
	Weighted median	16	1.11 (1.03–1.20)	.0044	–	–	–	–	
	Weighted mode	16	1.12 (1.04–1.20)	.0063	–	–	–	–	
5alpha-androstan-3beta,17beta-diol disulfate	IVW	16	1.11 (1.02–1.21)	.0157	8.5173	.9014	-0.0156	0.1676	.7210
	MR Egger	16	1.18 (1.05–1.37)	.0166	6.3994	.9554	–	–	
	Simple mode	16	1.09 (0.85–1.40)	.4929	–	–	–	–	
	Weighted median	16	1.17 (1.05–1.29)	.0036	–	–	–	–	
	Weighted mode	16	1.17 (1.06–1.29)	.0073	–	–	–	–	
5alpha-androstan-3beta,17beta-diol monosulfate (2)	IVW	21	1.10 (1.03–1.16)	.0025	10.6590	.9546	−0.0126	0.1750	.9533
	MR Egger	21	1.13 (1.05–1.22)	.0040	8.6742	.9785	–	–	
	Simple mode	21	1.06 (0.85–1.31)	.6092	–	–	–	–	
	Weighted median	21	1.10 (1.03–1.18)	.0031	–	–	–	–	
	Weighted mode	21	1.10 (1.03–1.18)	.0100	–	–	–	–	
5alpha-androstan-3alpha,17beta-diol monosulfate (1)	IVW	18	1.06 (1.00–1.17)	.0452	22.2964	.1736	−0.0189	0.1013	.2847
	MR Egger	18	1.10 (1.03–1.17)	.0146	18.7538	.2816	–	–	
	Simple mode	18	1.07 (0.91–1.25)	.4162	–	–	–	–	
	Weighted median	18	1.08 (1.03–1.14)	.0024	–	–	–	–	
	Weighted mode	18	1.09 (1.03–1.14)	.0066	–	–	–	–	
16a-hydroxy DHEA 3-sulfate	IVW	19	0.91 (0.86–0.97)	.0043	19.3727	.3692	0.0006	0.9442	.4690
	MR Egger	19	0.91 (0.84–0.99)	.0361	19.3670	.3079	–	–	
	Simple mode	19	0.90 (0.74–1.09)	.2920	–	–	–	–	
	Weighted median	19	0.90 (0.84–0.97)	.0045	–	–	–	–	
	Weighted mode	19	0.90 (0.84–0.97)	.0106	–	–	–	–	
Androstenediol (3beta,17beta) monosulfate (2)	IVW	19	1.20 (1.05–1.37)	.0059	24.6643	.1344	−0.0179	0.2902	.1587
	MR Egger	19	1.39 (1.04–1.86)	.0413	23.0482	.1477	–	–	
	Simple mode	19	1.28 (0.96–1.70)	.1104	–	–	–	–	
	Weighted median	19	1.36 (1.16–1.59)	.0001	–	–	–	–	
	Weighted mode	19	1.34 (1.12–1.61)	.0053	–	–	–	–	
1-stearoyl-2-linoleoyl-gpc (18:0/18:2)	IVW	16	0.79 (0.70–0.89)	.0000	13.7732	.5428	0.0143	0.3657	.6420
	MR Egger	16	0.70 (0.54–0.92)	.0222	12.8991	.5345	–	–	
	Simple mode	16	0.79 (0.62–1.00)	.0712	–	–	–	–	
	Weighted median	16	0.77 (0.65–0.90)	.0011	–	–	–	–	
	Weighted mode	16	0.77 (0.66–0.90)	.0055	–	–	–	–	
1-(1-enyl-palmitoyl)-2-arachidonoyl-gpc (*P*-16:0/20:4)	IVW	23	1.12 (1.05–1.19)	.0005	16.4153	.7948	−0.0029	0.7761	.8150
	MR Egger	23	1.13 (1.02–1.26)	.0261	16.3323	.7507	–	–	
	Simple mode	23	1.11 (0.92–1.34)	.2916	–	–	–	–	
	Weighted median	23	1.14 (1.04–1.24)	.0026	–	–	–	–	
	Weighted mode	23	1.13 (1.04–1.22)	.0065	–	–	–	–	
Arachidonate (20:4n6)	IVW	13	1.15 (1.04–1.27)	.0047	10.2257	.5962	−0.0172	0.3077	.5313
	MR Egger	13	1.25 (1.04–1.50)	.0339	9.0813	.6144			
	Simple mode	13	1.19 (0.91–1.55)	.2368					
	Weighted median	13	1.22 (1.08–1.38)	.0013					
	Weighted mode	13	1.22 (1.07–1.38)	.0099					
Cysteinylglycine	IVW	11	0.87 (0.78–0.98)	.0185	9.7324	.4643	0.0247	0.1913	.4430
	MR Egger	11	0.74 (0.57–0.96)	.0480	7.7355	.5610		–	
	Simple mode	11	0.88 (0.69–1.14)	.3622	–	–	–	–	
	Weighted median	11	0.80 (0.69–0.92)	.0022	–	–	–	–	
	Weighted mode	11	0.81 (0.69–0.95)	.0246	–	–	–	–	
Arachidonate (20:4n6) to linoleate (18:2n6) ratio	IVW	12	1.14 (1.04–1.23)	.0032	10.7623	.4634	−0.0060	0.6319	.5320
	MR Egger	12	1.16 (1.02–1.33)	.0469	10.5057	.3973	–	–	
	Simple mode	12	1.16 (0.93–1.46)	.2230	–	–	–	–	
	Weighted median	12	1.16 (1.06–1.27)	.0019	–	–	–	–	
	Weighted mode	12	1.16 (1.06–1.27)	.0063	–	–	–	–	

IVW = inverse-variance weighted, MR = Mendelian randomization, OR = odds ratio, SNP = single nucleotide polymorphism.

#### 3.2.2. Reverse MR analysis

Subsequently, reverse MR analysis was carried out to evaluate the influence of polymyalgia rheumatica on the 12 metabolites and 1 metabolite ratio identified in the forward MR analysis. SNPs with a significance threshold of *P* <5 × 10⁻⁸ were chosen as genetic instruments closely associated with polymyalgia rheumatica. The IVW method revealed that polymyalgia rheumatica could significantly elevate the level of Cysteinylglycine (OR = 1.14, 95%CI = 1.04–1.24, P_IVW_ = 0.007) (Table S1, Supplemental Digital Content, https://links.lww.com/MD/P871).

### 3.3. Heterogeneity Analysis and Pluripotency Analysis

**Figure 2.**
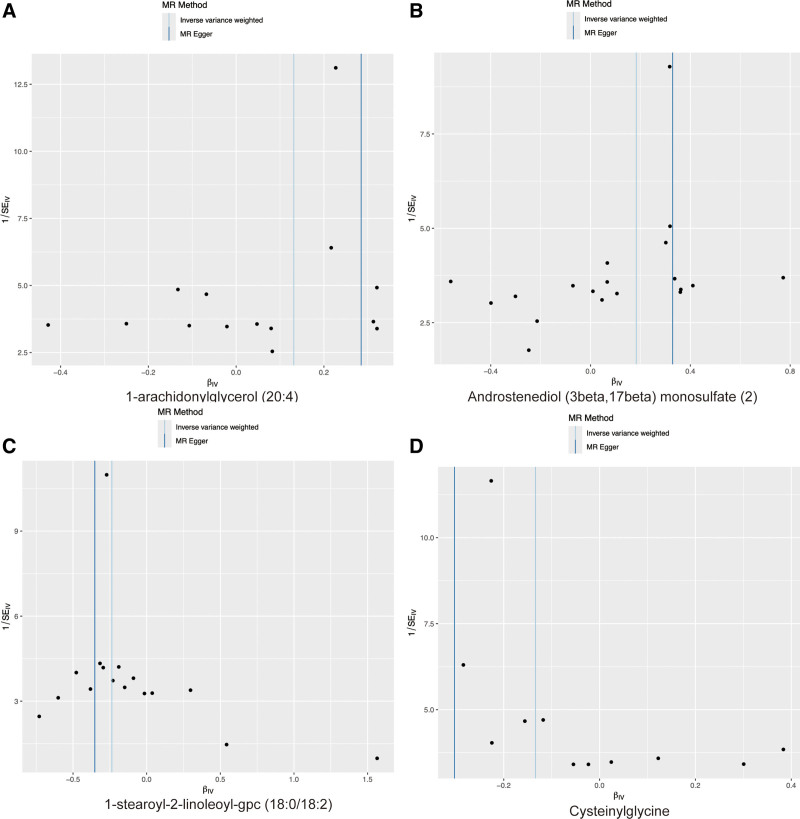
Representative funnel plot of 13 metabolites with potential causal relationship with PMR. IVW test generated P values of 0.4498 (A), 0.1344 (B), 0.5428 (C), and 0.4643 (D), respectively, which were consistent with those arising from Egger test, indicated that there was no evidence of heterogeneity. IVW = inverse-variance weighted, PMR = polymyalgia rheumatic.

The results of the heterogeneity and pluripotency analyses are presented in Table 1. The findings suggested that there was no significant heterogeneity in the IVW and MR Egger methods. Additionally, as depicted in the funnel plot (Fig. 2; Fig. S3, Supplemental Digital Content, https://links.lww.com/MD/P870), there was no obvious symmetry in the variation of the effect size around the estimated point. Horizontal pleiotropy was evaluated through the intercept of the MR-Egger method and MR-PRESSO. The intercept of MR-Egger did not significantly deviate from the zero intercept of IVW, indicating the absence of horizontal pluripotency. Additionally, MR-PRESSO analysis revealed no evidence of horizontal pluripotency.

### 3.4. Leave-one-out analysis

**Figure 3.**
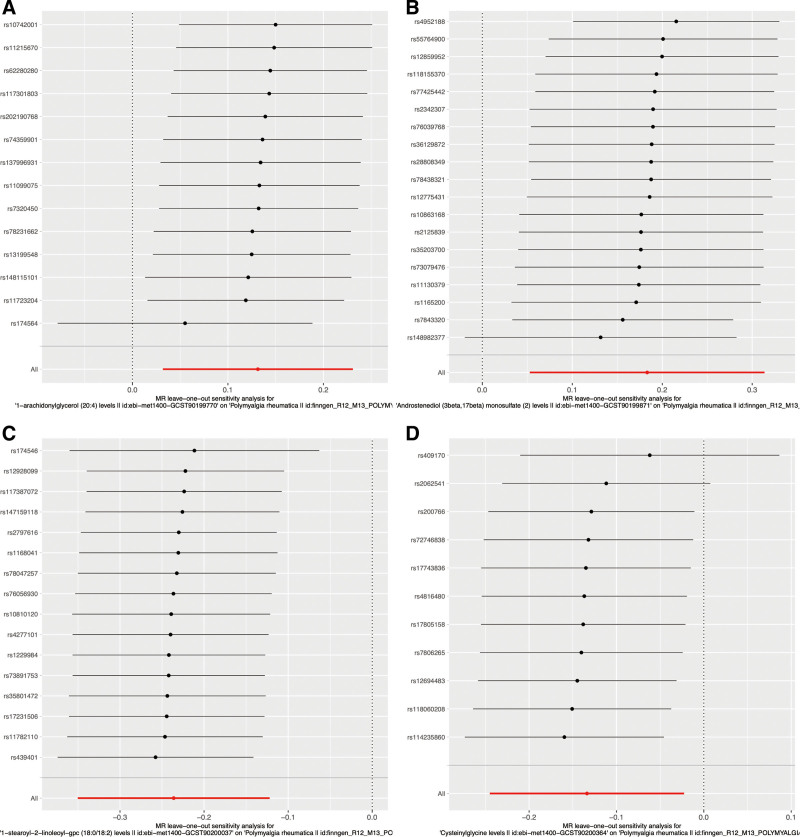
Representative leave-one-out forest maps of 13 metabolites with potential causal relationship with PMR. The overall error line was not significantly impacted by the elimination of each individual SNP. As a result, the correlation analysis outcomes of the 2-sample MR study were fairly stable. MR = Mendelian randomization, PMR = polymyalgia rheumatic, SNP = single nucleotide polymorphism.

A leave-one-out analysis was conducted to calculate the MR results of the remaining IVs when each one was removed separately. As shown in Figure 3; Figure S4, Supplemental Digital Content, https://links.lww.com/MD/P870, the removal of each SNP did not notably affect the overall error line. Thus, the correlation analysis results from the 2-sample MR study were relatively stable.

## 4. Discussion

In this bidirectional comparison involving 2 sets of samples, a suggestive association between PMR and the levels of certain serum metabolites was detected. By using genetic variants as probes, we identified 13 metabolites associated with the risk of PMR at a significance level of *P* <.05. Specifically, 10 of these serum metabolites significantly increased the risk of PMR (*P* <.05), while 3 significantly decreased the risk (*P* <.05). Additionally, our study revealed 1 blood metabolite with bidirectional effects.

We identified 5 androgen-related metabolites, namely Epiandrosterone sulfate, 5alpha-androstan-3beta, 17beta-diol disulfate, 5alpha-androstan-3beta,17beta-diol monosulfate (2), 5alpha-androstan-3alpha, 17beta-diol monosulfate (1), and Androstenediol (3beta,17beta) monosulfate (2), as causal risk factors for PMR. However, another androgen-related metabolite, 16a-hydroxy DHEA 3-sulfate, was found to reduce the risk of PMR.

PMR is a chronic inflammatory disease characterized by pain and morning stiffness in the muscles of the shoulders, neck, and pelvic girdle. It is often accompanied by a systemic inflammatory response and elevated erythrocyte sedimentation rate and C-reactive protein.^[17]^ Androgens have been shown to significantly suppress the level of inflammation in the body. In patients with active and untreated PMR, an increase in androstenedione and dehydroepiandrosterone sulphate serum levels has been reported, which is considered one of the important causes of PMR.^[18–21]^ This phenomenon may be related to reduced adrenal function^[22,23]^ (Javier, 2006 #73).

Our study shows that 5 androgen-related metabolites can promote the development of PMR, while 1 androgen-related metabolite can inhibit it. This indicates that the impact of androgen-related metabolites on PMR is complex. Moreover, due to the complex forms and metabolism of androgens in the body, which are not yet fully understood, further research is needed to clarify the metabolic process of androgens in the body and prove the specific role of androgen-related metabolites in the pathogenesis of PMR.

In addition, we found that 4 arachidonic acid (AA)-related metabolites and 1 ratio of AA-related metabolites – 1-arachidonylglycerol (20:4), 1-arachidonoyl-gpc (20:4n6), 1-(1-enyl-palmitoyl)-2-arachidonoyl-gpc (*P*-16:0/20:4), the ratio of arachidonate (20:4n6) to linoleate (18:2n6), and arachidonate (20:4n6) – increase the risk of PMR. As a polyunsaturated fatty acid (PUFA), AA is a key component of mammalian cell membranes, released by phospholipase A2, and serves critical roles in maintaining membrane structure and function.^[24]^ As a well-known proinflammatory molecule, AA triggers oxidative stress and immune responses, making intervention in its metabolic pathway a validated clinical strategy for managing inflammatory diseases.^[24–27]^ Given that PMR is an inflammatory disorder, the promotion of PMR pathogenesis by AA-related metabolites is plausible. Notably, our study is the first to report this association, though experimental validation is needed.

In MR analysis of rheumatoid arthritis (RA), another common immune disease, PUFAs also influence RA risk. A higher ratio of PUFAs to total fatty acids significantly correlates with increased RA risk.^[28]^ PUFAs generate proinflammatory mediators like prostaglandin E2 via the cyclooxygenase pathway, promoting synovial cell proliferation, angiogenesis, neutrophil infiltration, and exacerbating joint inflammation and bone destruction.^[28,29]^ PUFA metabolites also activate the NF-κB signaling pathway, inducing release of proinflammatory cytokines and driving Th1/Th17 cell polarization, thus establishing a “metabolism-immunity” vicious cycle.^[29]^ AA-related metabolites may also promote PMR through this signaling pathway.

Our study also showed that 1-stearoyl – 2-linoleoyl-gpc (18:0/18:2) promotes the development of PMR. Currently, there are few studies on 1-stearoyl-2-linoleoyl-gpc (18:0/18:2). Yixi Sun et al reported that the serum levels of 1-stearoyl-2-linoleoyl-gpc (18:0/18:2) vary in people consuming different diets, and this difference may ultimately affect the blood pressure levels of the subjects.^[30]^ At present, the function of 1-stearoyl-2-linoleoyl-gpc (18:0/18:2) is unclear, and its specific impact on PMR requires further research.

Cysteinylglycine, a sulfhydryldipeptide, is produced from extracellular glutathione, a non-protein thiol, through the catalytic activity of γ–glutamyltransferase.^[31]^ Our analysis showed that Cysteinylglycine reduces the risk of PMR, while PMR conversely increases the levels of Cysteinylglycine metabolites in serum, indicating a complex role of Cysteinylglycine in PMR. There are also limited studies on Cysteinylglycine. It has been found to be associated with tumors and may promote the occurrence of various malignant tumors.^[32–34]^ The interaction between Cysteinylglycine and PMR requires more in-depth research in the future.

Our findings should be interpreted with caution due to several limitations. First, the predominantly European ancestry of the GWAS data may limit the generalizability of our results to other populations. Thus, validation in diverse ethnic groups is necessary. Second, although our analysis covered a wide metabolomic profile, the functional roles and mechanistic links of certain metabolites to PMR remain unclear, which restricts causal interpretation. Further research is warranted to address these limitations and gain a more comprehensive understanding of the relationship between metabolites and PMR. Third, stratified analysis by age, sex, and comorbidities is critical for understanding the heterogeneity of PMR-metabolite relationships. The current study’s lack of such analysis limits its ability to address how these factors modify causal effects. Future research should prioritize subgroup analyses to enhance the clinical applicability of metabolic markers and guide personalized PMR prevention and treatment strategies. Fourth, Although MR minimizes confounding by leveraging genetic variants as IVs, it is important to acknowledge that nongenetic factors such as diet, medication use, and environmental exposures may still influence metabolite levels and indirectly affect the observed associations. These factors could introduce residual variability in the causal estimates, highlighting the need for future studies to incorporate detailed covariate adjustments or stratified analyses to further validate our findings.

## 5. Conclusion

This bidirectional MR study identified 13 serum metabolites causally linked to PMR, including androgen- and AA-related metabolites that promote PMR risk, and cysteinylglycine, which exhibits a bidirectional relationship. These findings highlight the role of inflammatory pathways and hormonal dysregulation in PMR pathogenesis, offering potential biomarkers and therapeutic targets. Despite limitations in population generalizability and mechanistic clarity, this study advances understanding of PMR’s metabolic underpinnings. Future research should validate these associations in diverse cohorts and elucidate functional mechanisms to guide metabolite-based interventions for this chronic inflammatory disorder.

Supplemental digital contents “IVs for forward MR and IVs for reverse MR” are available for this article (https://links.lww.com/MD/P872, https://links.lww.com/MD/P873)

## Author contributions

**Conceptualization:** yafang wang, Huiyun Li.

**Data curation:** Aifeng Song, Lin Kang, Saixia Cao.

**Investigation:** Aifeng Song, Lin Kang, Saixia Cao.

**Methodology:** yafang wang, Huiyun Li.

**Resources:** yafang wang, Huiyun Li.

**Supervision:** yafang wang, Huiyun Li.

**Validation:** yafang wang, Huiyun Li.

**Writing – original draft:** Aifeng Song, Lin Kang.

**Writing – review & editing:** Aifeng Song, Lin Kang.

## Supplementary Material


