# Supplementary material for: Bidirectional Mendelian randomization study reveals causal relationships between polymyalgia rheumatica and serum metabolites
Source: Medicine (Baltimore). 2025 Sep 5;104(36):e44304. doi: 10.1097/MD.0000000000044304 (PMC12419372; doi:10.1097/MD.0000000000044304)
Supplement: Supplementary file 1 [file medi-104-e44304-s001.pdf]

Figure S1 The overview of the research work flow.

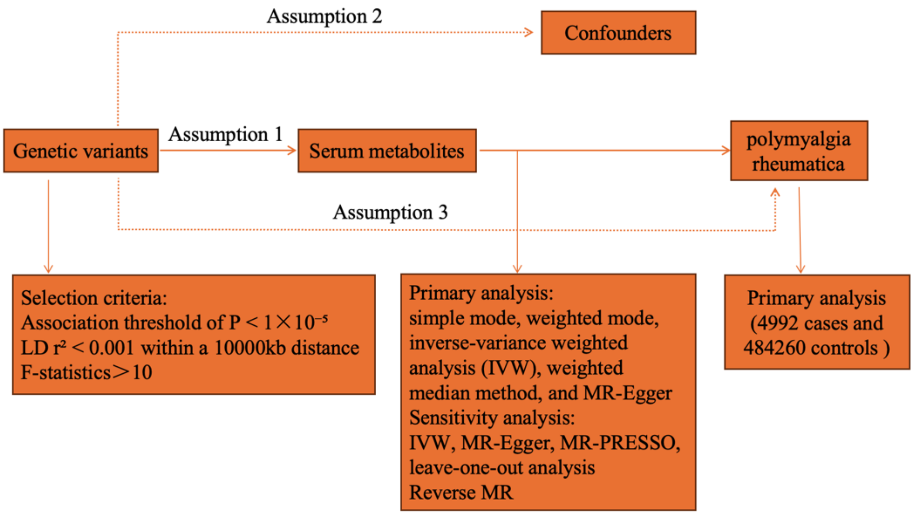

Figure S2 Scatter plots of the 5MR models for other 9 screened metabolites with potential causal relationship with PMR. MR, Mendelian randomization; SNP, single nucleotide polymorphism; PMR, polymyalgia rheumatica.

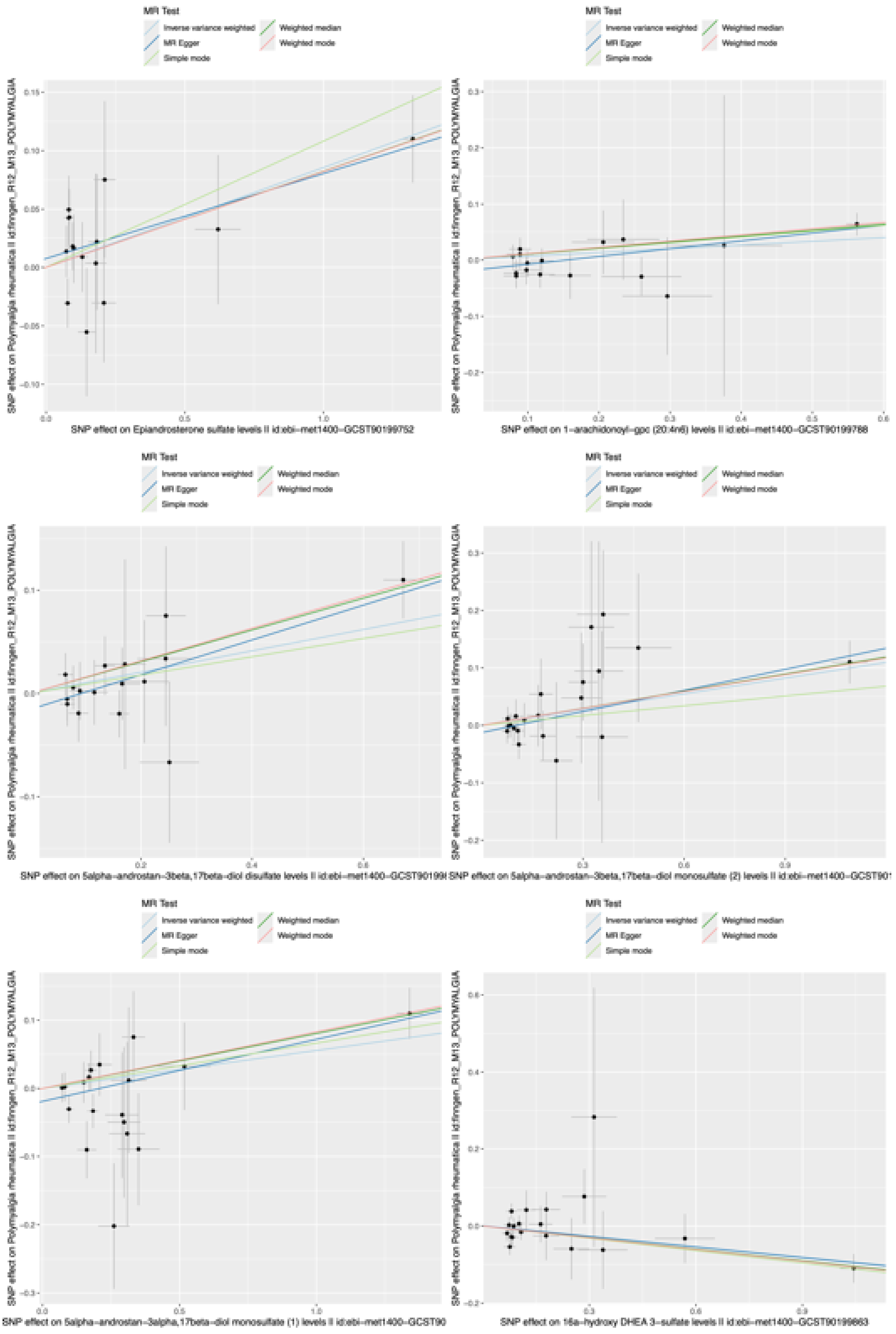

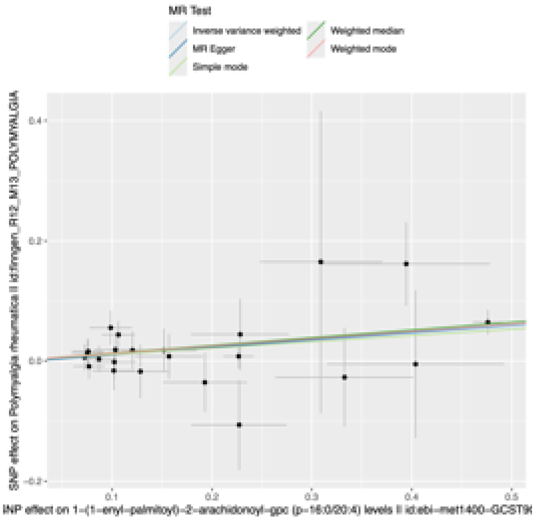
MR Test
Inverse variance weighted
MR Egger
Simple mode
Weighted median
Weighted mode
SNP effect on Polymyalgia rheumatica || id:finngen_R12_M13_POLYMYALGIA
0.4
0.2
0.0
-0.2
0.1
0.2
0.3
0.4
0.5
INP effect on 1-(1-enyl-palmitoyl)-2-arachidonoyl-gpc (p-16:0/20:4) levels || id:ebi-met1400-GCST9

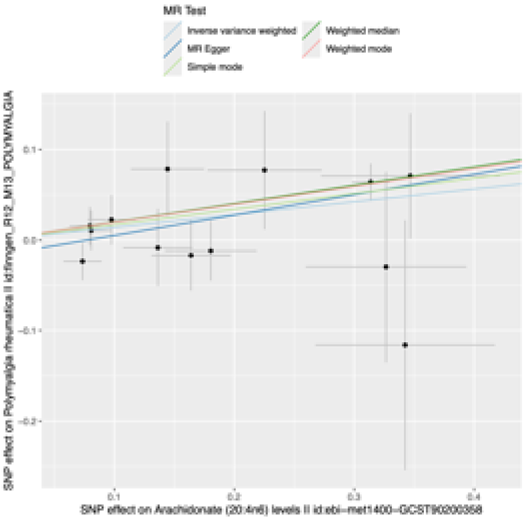
MR Test
Inverse variance weighted
MR Egger
Simple mode
Weighted median
Weighted mode
SNP effect on Polymyalgia rheumatica || id:finngen_R12_M13_POLYMYALGIA
0.1
0.0
-0.1
-0.2
0.1
0.2
0.3
0.4
SNP effect on Arachidonate (20:4n6) levels || id:ebi-met1400-GCST90200358

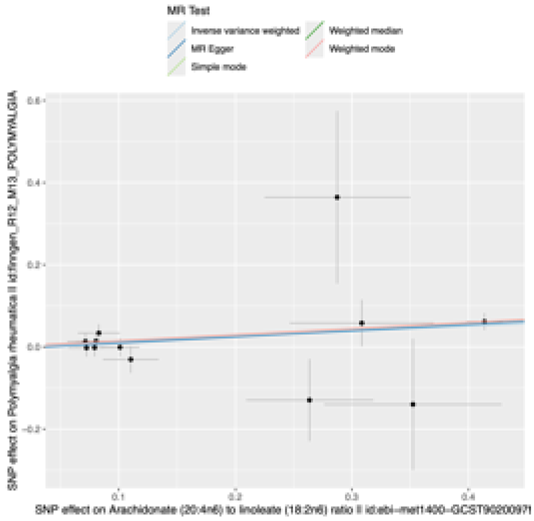
MR Test
Inverse variance weighted
MR Egger
Simple mode
Weighted median
Weighted mode
SNP effect on Polymyalgia rheumatica || id:finngen_R12_M13_POLYMYALGIA
0.6
0.4
0.2
0.0
-0.2
0.1
0.2
0.3
0.4
SNP effect on Arachidonate (20:4n6) to linoleate (18:2n6) ratio || id:ebi-met1400-GCST9020097

Figure S3 Funnel plot of other 9 metabolites with potential causal relationship with PMR. MR,Mendelian randomization; PMR, polymyalgia rheumatica.

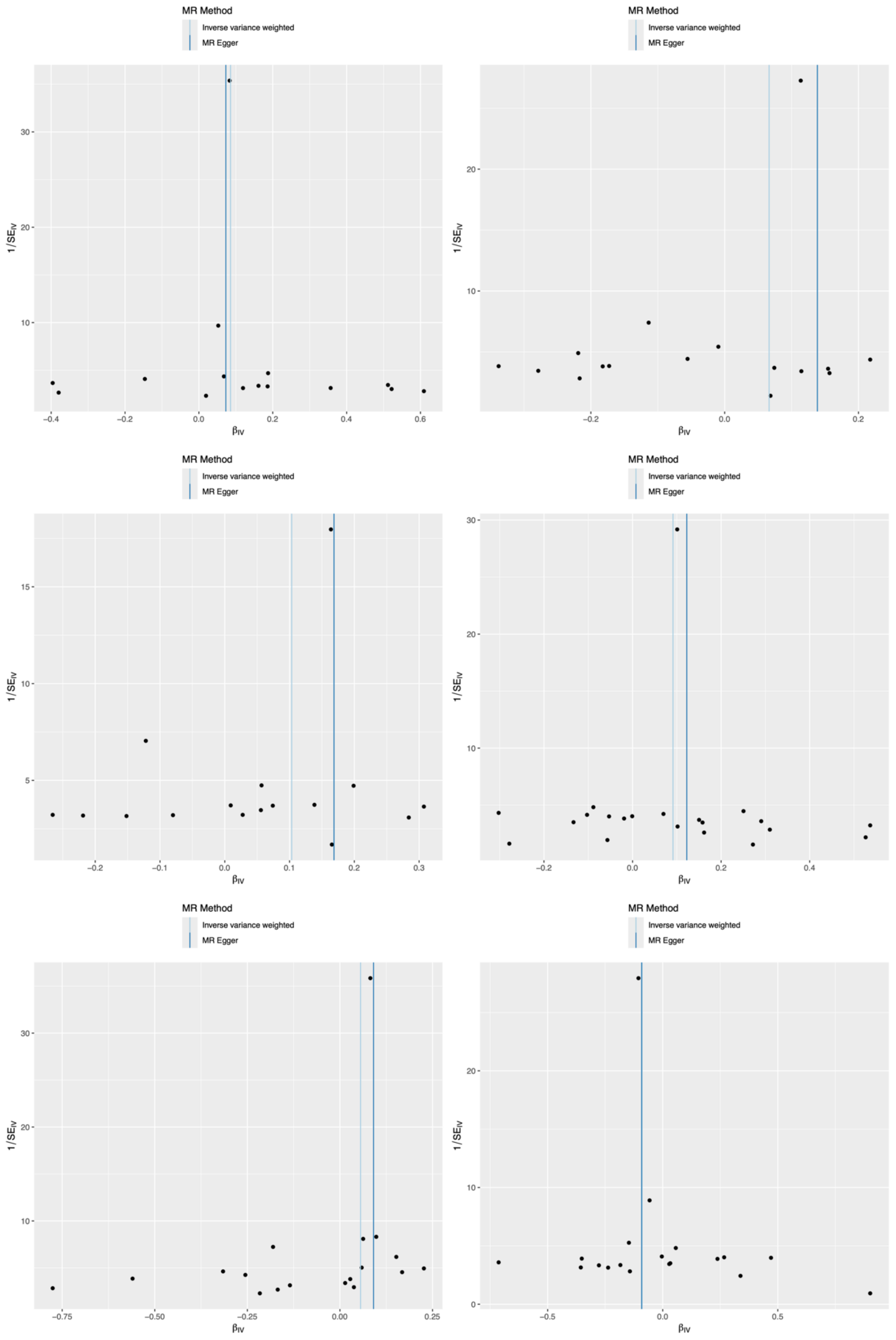

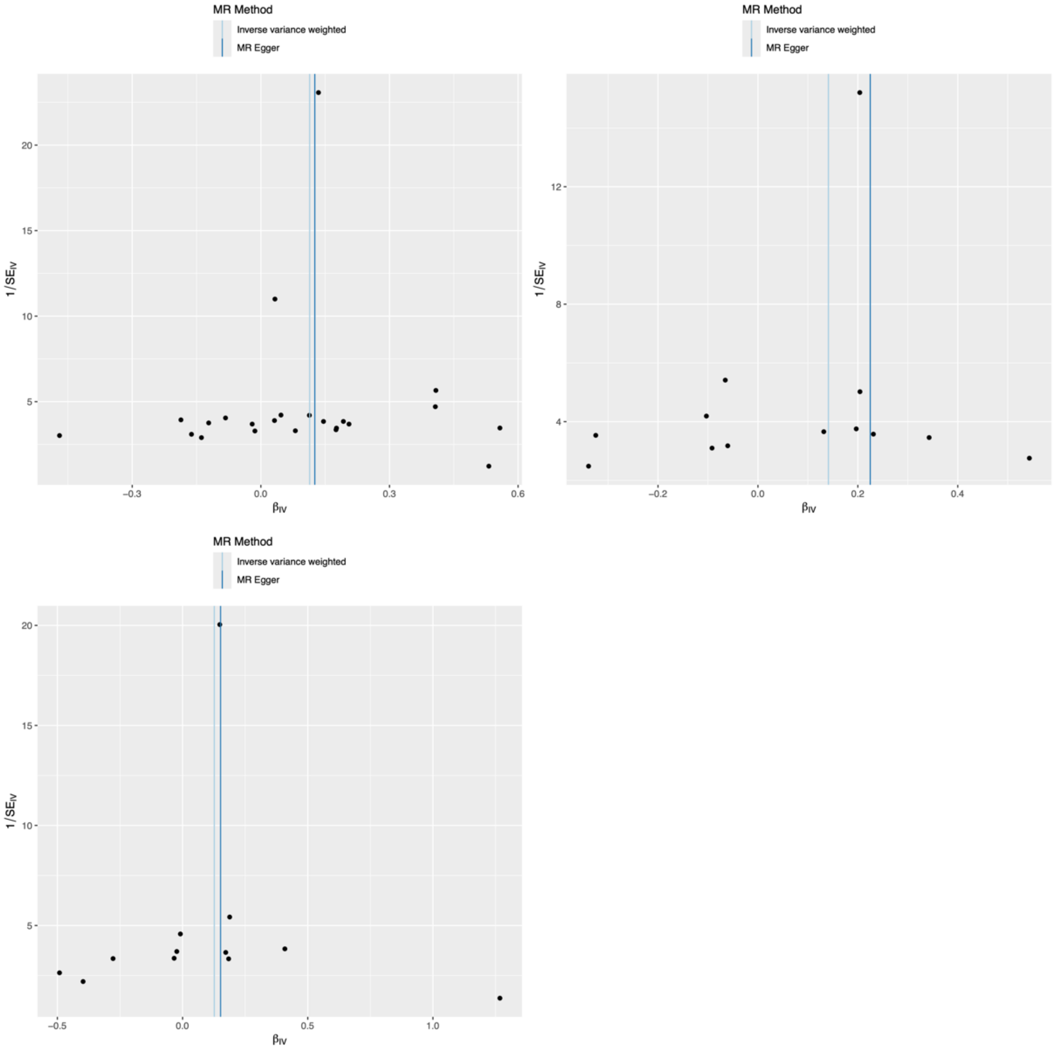
MR Method
Inverse variance weighted
MR Egger
1/SE$_{IV}$
β$_{IV}$
MR Method
Inverse variance weighted
MR Egger
1/SE$_{IV}$
β$_{IV}$
MR Method
Inverse variance weighted
MR Egger
1/SE$_{IV}$
β$_{IV}$

Figure S4 Leave-one-out forest maps of other 9 metabolites with potential causal relationship with PMR. MR, Mendelian randomization; SNP, single nucleotide polymorphism; PMR, polymyalgia rheumatica.

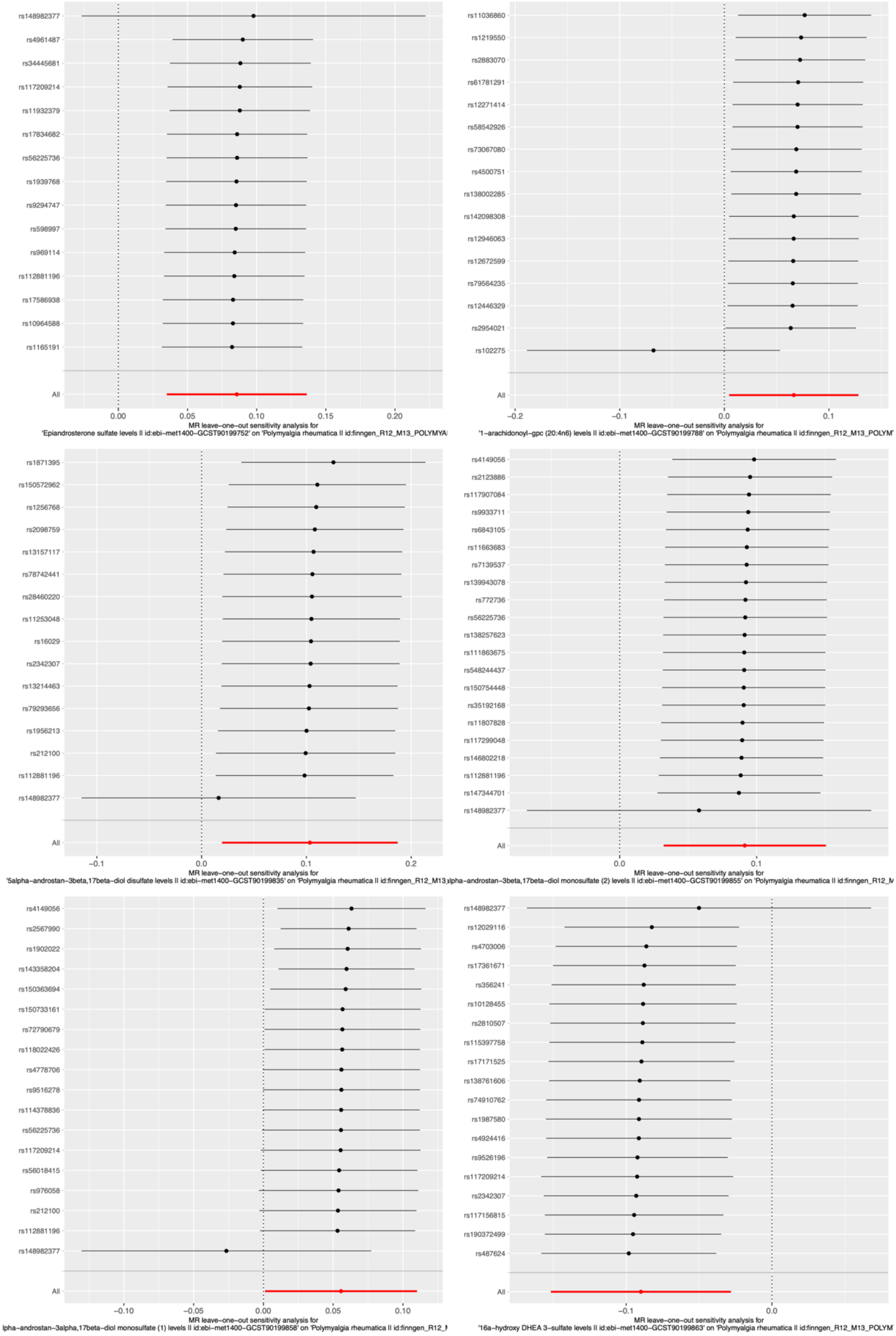

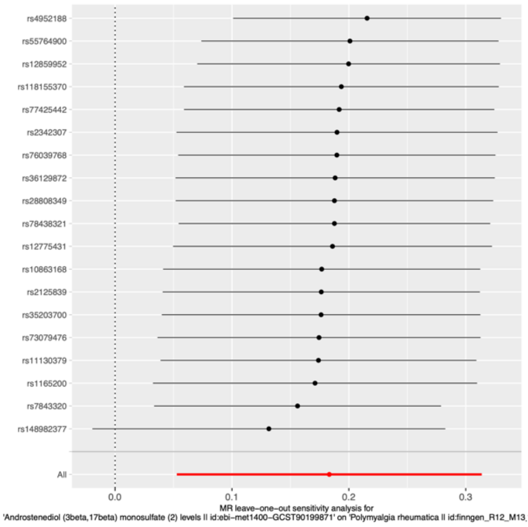
rs4952188
rs55764900
rs12859952
rs118155370
rs77425442
rs2342307
rs76039768
rs36129872
rs28808349
rs78438321
rs12775431
rs10863168
rs2125839
rs35203700
rs73079476
rs11130379
rs1165200
rs7843320
rs148982377
All
0.0
0.1
0.2
0.3
MR leave–one–out sensitivity analysis for
'Androstenediol (3beta,17beta) monosulfate (2) levels II id:ebi–met1400–GCST90199871' on 'Polymyalgia rheumatica II id:finngen_R12_M13

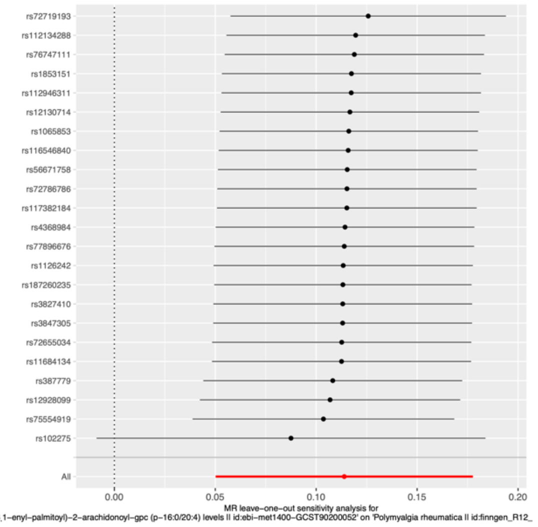
rs72719193
rs112134288
rs76747111
rs1853151
rs112946311
rs12130714
rs1065853
rs116546840
rs56671758
rs72786786
rs117382184
rs4368984
rs77896676
rs1126242
rs187260235
rs3827410
rs3847305
rs72655034
rs11684134
rs387779
rs12928099
rs75554919
rs102275
All
0.00
0.05
0.10
0.15
0.20
MR leave–one–out sensitivity analysis for
1–enyl–palmitoyl)–2–arachidonoyl–gpc (p–16:0/20:4) levels II id:ebi–met1400–GCST90200052' on 'Polymyalgia rheumatica II id:finngen_R12_

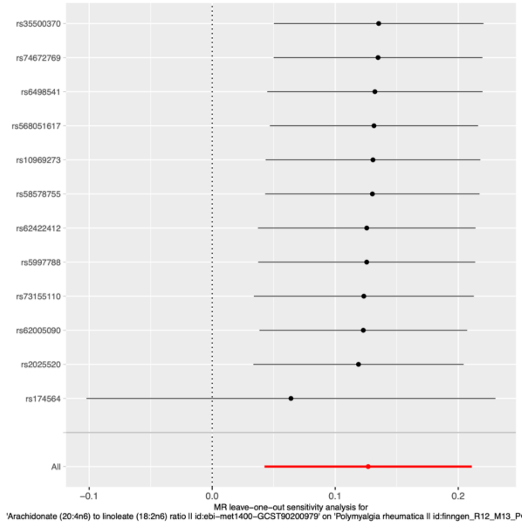
rs35500370
rs74672769
rs6498541
rs568051617
rs10969273
rs58578755
rs62422412
rs5997788
rs73155110
rs62005090
rs2025520
rs174564
All
−0.1
0.0
0.1
0.2
MR leave–one–out sensitivity analysis for
'Arachidonate (20:4n6) to linoleate (18:2n6) ratio II id:ebi–met1400–GCST90200979' on 'Polymyalgia rheumatica II id:finngen_R12_M13_P
