# Supplementary material for: Bidirectional Mendelian randomization study reveals causal relationships between polymyalgia rheumatica and serum metabolites
Source: Medicine (Baltimore). 2025 Sep 5;104(36):e44304. doi: 10.1097/MD.0000000000044304 (PMC12419372; doi:10.1097/MD.0000000000044304)
Supplement: Supplementary file 2 [file medi-104-e44304-s002.docx]

Table S1 Complementary reverse MR analyses of polymyalgia rheumatica for causal association with metabolites and tests for heterogeneity and horizontal pleiotropy. MR, Mendelian randomization; IVW, inverse-variance weighted.

|  | MR analysis | | | | Heterogeneity | | Pleiotropy | | |
| --- | --- | --- | --- | --- | --- | --- | --- | --- | --- |
| Metabolites | Methods | SNP(N) | OR(95% CI) | *p* | Q value | *P* | Intercept | *P* _MR Egger_ | *P* _MR-PRESSO_ |
| Cysteinylglycine | IVW | 4 | 1.14(1.04-1.24) | 0.0067 | 5.4068 | 0.14432 | -0.0646 | 0.1604 |  |
|  | MR Egger | 4 | 1.41(1.15-1.74) | 0.0826 | 0.6277 | 0.73063 |  |  |  |
|  | Simple mode | 4 | 1.17(0.99-1.37) | 0.1598 |  |  |  |  |  |
|  | Weighted median | 4 | 1.18(1.09-1.28) | 0.0000 |  |  |  |  |  |
|  | Weighted mode | 4 | 1.18(1.09-1.29) | 0.0290 |  |  |  |  |  |
| 5alpha-androstan-3alpha,17beta-diol monosulfate (1) | IVW | 4 | 0.98(0.92-1.05) | 0.6273 | 1.4376 | 0.69675 | 0.0002 | 0.9953 |  |
|  | MR Egger | 4 | 0.98(0.80-1.21) | 0.8835 | 1.4375 | 0.48735 |  |  |  |
|  | Simple mode | 4 | 1.00(0.89-1.11) | 0.9475 |  |  |  |  |  |
|  | Weighted median | 4 | 0.99(0.92-1.07) | 0.8156 |  |  |  |  |  |
|  | Weighted mode | 4 | 0.99(0.91-1.08) | 0.8499 |  |  |  |  |  |
| 16a-hydroxy DHEA 3-sulfate | IVW | 4 | 0.97(0.91-1.04) | 0.3754 | 0.9643 | 0.8099 | 0.0002 | 0.9961 |  |
|  | MR Egger | 4 | 0.97(0.79-1.19) | 0.7950 | 0.9642 | 0.61748 |  |  |  |
|  | Simple mode | 4 | 0.97(0.87-1.09) | 0.6646 |  |  |  |  |  |
|  | Weighted median | 4 | 0.97(0.90-1.04) | 0.3849 |  |  |  |  |  |
|  | Weighted mode | 4 | 0.97(0.89-1.05) | 0.5129 |  |  |  |  |  |
| Androstenediol (3beta,17beta) monosulfate (2) | IVW | 4 | 0.96(0.89-1.03) | 0.2427 | 3.6934 | 0.29653 | 0.0055 | 0.9023 |  |
|  | MR Egger | 4 | 0.94(0.71-1.24) | 0.6994 | 3.6581 | 0.16056 |  |  |  |
|  | Simple mode | 4 | 0.95(0.84-1.09) | 0.5326 |  |  |  |  |  |
|  | Weighted median | 4 | 0.95(0.88-1.02) | 0.1829 |  |  |  |  |  |
|  | Weighted mode | 4 | 0.95(0.88-1.03) | 0.2997 |  |  |  |  |  |
| 1-stearoyl-2-linoleoyl-gpc (18:0/18:2) | IVW | 4 | 1.02(0.95-1.09) | 0.5831 | 2.2404 | 0.52403 | -0.0115 | 0.7349 |  |
|  | MR Egger | 4 | 1.06(0.86-1.30) | 0.6411 | 2.0829 | 0.35294 |  |  |  |
|  | Simple mode | 4 | 1.04(0.91-1.18) | 0.6084 |  |  |  |  |  |
|  | Weighted median | 4 | 1.04(0.96-1.12) | 0.3411 |  |  |  |  |  |
|  | Weighted mode | 4 | 1.04(0.96-1.13) | 0.3876 |  |  |  |  |  |
| 1-arachidonoyl-gpc (20:4n6) | IVW | 4 | 0.99(0.92-1.07) | 0.8447 | 3.3654 | 0.33865 | -0.0049 | 0.91 |  |
|  | MR Egger | 4 | 1.01(0.77-1.32) | 0.9520 | 3.3381 | 0.18842 |  |  |  |
|  | Simple mode | 4 | 0.99(0.88-1.12) | 0.9407 |  |  |  |  |  |
|  | Weighted median | 4 | 1.00(0.93-1.08) | 0.9932 |  |  |  |  |  |
|  | Weighted mode | 4 | 1.00(0.92-1.09) | 0.9870 |  |  |  |  |  |
| Epiandrosterone sulfate | IVW | 4 | 1.01(0.94-1.08) | 0.8806 | 3.3119 | 0.34599 | 0.0068 | 0.867 |  |
|  | MR Egger | 4 | 0.98(0.76-1.27) | 0.9024 | 3.2533 | 0.19658 |  |  |  |
|  | Simple mode | 4 | 1.05(0.95-1.17) | 0.4158 |  |  |  |  |  |
|  | Weighted median | 4 | 1.02(0.95-1.09) | 0.6516 |  |  |  |  |  |
|  | Weighted mode | 4 | 1.01(0.94-1.09) | 0.7930 |  |  |  |  |  |
| 5alpha-androstan-3beta,17beta-diol monosulfate (2) | IVW | 4 | 0.99(0.89-1.09) | 0.7808 | 6.0649 | 0.1085 | -0.0357 | 0.5188 |  |
|  | MR Egger | 4 | 1.11(0.80-1.54) | 0.5833 | 4.6608 | 0.09726 |  |  |  |
|  | Simple mode | 4 | 1.01(0.92-1.12) | 0.8073 |  |  |  |  |  |
|  | Weighted median | 4 | 1.02(0.95-1.10) | 0.5927 |  |  |  |  |  |
|  | Weighted mode | 4 | 1.02(0.94-1.10) | 0.6569 |  |  |  |  |  |
| 5alpha-androstan-3beta,17beta-diol disulfate | IVW | 4 | 1.00(0.93-1.07) | 0.9779 | 3.8583 | 0.27717 | -0.0214 | 0.5714 |  |
|  | MR Egger | 4 | 1.07(0.86-1.35) | 0.5955 | 3.1495 | 0.20706 |  |  |  |
|  | Simple mode | 4 | 0.94(0.83-1.05) | 0.3472 |  |  |  |  |  |
|  | Weighted median | 4 | 1.00(0.93-1.07) | 0.9745 |  |  |  |  |  |
|  | Weighted mode | 4 | 1.00(0.93-1.08) | 0.9308 |  |  |  |  |  |
| 1-(1-enyl-palmitoyl)-2-arachidonoyl-gpc (p-16:0/20:4) | IVW | 4 | 1.05(0.98-1.12) | 0.1950 | 2.2896 | 0.51451 | 0.0318 | 0.3962 |  |
|  | MR Egger | 4 | 0.94(0.76-1.16) | 0.6169 | 1.1423 | 0.56487 |  |  |  |
|  | Simple mode | 4 | 1.09(0.95-1.24) | 0.2990 |  |  |  |  |  |
|  | Weighted median | 4 | 1.04(0.96-1.13) | 0.3071 |  |  |  |  |  |
|  | Weighted mode | 4 | 1.01(0.94-1.10) | 0.7514 |  |  |  |  |  |
| Arachidonate (20:4n6) to linoleate (18:2n6) ratio | IVW | 4 | 0.98(0.88-1.10) | 0.7760 | 7.2793 | 0.06351 | -0.0470 | 0.4133 |  |
|  | MR Egger | 4 | 1.16(0.84-1.60) | 0.4744 | 4.7736 | 0.09192 |  |  |  |
|  | Simple mode | 4 | 0.90(0.77-1.06) | 0.3059 |  |  |  |  |  |
|  | Weighted median | 4 | 0.98(0.91-1.06) | 0.6982 |  |  |  |  |  |
|  | Weighted mode | 4 | 1.00(0.92-1.08) | 0.9469 |  |  |  |  |  |
| 1-arachidonylglycerol (20:4) | IVW | 4 | 1.01(0.93-1.10) | 0.7320 | 4.3406 | 0.22696 | 0.0476 | 0.2515 |  |
|  | MR Egger | 4 | 0.86(0.70-1.07) | 0.3036 | 1.7927 | 0.40805 |  |  |  |
|  | Simple mode | 4 | 0.98(0.84-1.14) | 0.8286 |  |  |  |  |  |
|  | Weighted median | 4 | 1.00(0.92-1.08) | 0.9283 |  |  |  |  |  |
|  | Weighted mode | 4 | 0.98(0.90-1.06) | 0.6560 |  |  |  |  |  |
| Arachidonate (20:4n6) | IVW | 4 | 0.98(0.92-1.05) | 0.5756 | 2.1417 | 0.54352 | 0.0201 | 0.5647 |  |
|  | MR Egger | 4 | 0.92(0.74-1.13) | 0.4940 | 1.6741 | 0.43299 |  |  |  |
|  | Simple mode | 4 | 0.96(0.86-1.07) | 0.5558 |  |  |  |  |  |
|  | Weighted median | 4 | 0.97(0.90-1.05) | 0.4218 |  |  |  |  |  |
|  | Weighted mode | 4 | 0.96(0.89-1.05) | 0.4744 |  |  |  |  |  |
